# Multi‐Omic Analysis of *Scylla serrata* Reveals the Allergen Landscape of Mud Crabs and Decapoda Species

**DOI:** 10.1111/all.70053

**Published:** 2025-09-09

**Authors:** Kelvin Fu‐Kiu Ao, Judy Kin Wing Ng, Cherie Tsz‐Yiu Law, Weixue Mu, Shanshan Li, Christine Yee Yan Wai, Man Fung Tang, Shannon Wing Ngor Au, Xiaoyu Liu, Ting Fan Leung, Qing Xiong, Stephen Kwok‐Wing Tsui

**Affiliations:** ^1^ School of Biomedical Sciences, Faculty of Medicine The Chinese University of Hong Kong Sha Tin Hong Kong SAR China; ^2^ Hong Kong Bioinformatics Centre The Chinese University of Hong Kong Sha Tin Hong Kong SAR China; ^3^ School of Life Sciences, Faculty of Science The Chinese University of Hong Kong Sha Tin Hong Kong SAR China; ^4^ Department of Paediatrics The Chinese University of Hong Kong Sha Tin Hong Kong SAR China; ^5^ Hong Kong Hub of Paediatric Excellence The Chinese University of Hong Kong Sha Tin Hong Kong SAR China; ^6^ Shenzhen Key Laboratory of Allergy and Immunology School of Medicine, Shenzhen University Shenzhen China; ^7^ Department of Health Technology and Informatics The Hong Kong Polytechnic University Hung Hom Hong Kong SAR China; ^8^ Centre for Microbial Genomics and Proteomics The Chinese University of Hong Kong Sha Tin Hong Kong SAR China

**Keywords:** allergens and epitopes, bioinformatics, food allergy, IgE, “omics” and “systems biology”

To the Editor,

*Scylla* (*S*.) *serrata* (giant mud crab) is commercially valued yet poses health risks by its allergenic potential, characterized by adult‐onset and Asia‐Pacific prevalence [1, 2]. Nonetheless, the absence of a complete allergen profile for crabs and *S. serrata* necessitates detailed molecular characterization. Current shellfish allergy diagnosis focuses on a subset of allergens, which may not reflect unique allergens in Asian‐consumed crabs [2]. Modern‐day allergen studies often combine genomics, transcriptomics, and proteomics for in silico allergen characterization with in vitro verification [3, 4].

Our multi‐omics study comprehensively analyzed allergenic components in *S. serrata* to understand the diverse Decapoda order and Arthropoda phylum [5]. Sequencing with three libraries (stLFR, ONT, Hi‐C) with the female crab has assembled a chromosome‐level genome of *S. serrata* containing 46 pseudo‐chromosomes (Figure S1, Table S1). Sequencing of two males confirmed the species identity (Table S2, Figure S2). The high completeness (92.2%) and continuity (N50 = 21,436,738 bp) illustrated the high‐quality genome characteristic (Table S3). The subsequent genome annotation of 26,643 protein‐coding genes demonstrated high proteome completeness (93.0%).

With the annotation building the BLAST database, the in silico allergen identification began with Decapoda allergen sequences in the WHO/IUIS Allergen Nomenclature as queries. We identified six known *S. serrata* allergens of the *Scylla* genus and seven novel allergens first appearing in the genus (Table S4). Their identity to published allergens reached 90% and 70% for known and novel allergens, respectively (Table 1, Figure S3). The comparative genomics analysis with seven Decapods revealed phylogenetic clusters of potential allergenic properties (Figure S4).

**TABLE 1:** Gene features of in silico predicted and manually curated known and putative allergen homologs in *Scylla serrata* .

Group	Gene locus	Biochemical function	# exons	# amino acids	Isoallergen ID	Accession number	Identity (%)
1	CR_022547.69	Tropomyosin	9	284	Scy p 1.0101	ABS12233.1	100
2	CR_015308.01	Arginine kinase	2	357	Scy p 2.0101	AFA45340.1	99.16
	CR_003142.02	Arginine kinase	1	361	Scy p 2.0101	AFA45340.1	51.55
3	CR_026644.02^a^	Myosin light chain 2	1	180	Lit v 3.0101	ACC76803.1	88.14
	CR_003127.02	Myosin light chain 2	1	177	Lit v 3.0101	ACC76803.1	65.14
	CR_004478.02	Myosin light chain 2	5	216	Lit v 3.0101	ACC76803.1	64.16
	CR_003927.01	Myosin light chain 2	2	144	Lit v 3.0101	ACC76803.1	27.66
	CR_005957.02	Myosin light chain 2	4	158	Lit v 3.0101	ACC76803.1	29.41
	CR_009145.01	Myosin light chain 2	4	172	Lit v 3.0101	ACC76803.1	24.22
	CR_015992.02	Myosin light chain 2	5	257	Lit v 3.0101	ACC76803.1	24.10
	CR_022614.02	Myosin light chain 2	3	153	Lit v 3.0101	ACC76803.1	22.67
4	CR_005380.03	Sarcoplasmic calcium‐binding protein	4	200	Scy p 4.0101	AFJ80778.1	90.67
5	CR_017542.02	Myosin light chain 1	5	154	Scy p 3.0101	QDH76468.1	96.73
6	CR_022928.02	Troponin C	4	122	Hom a 6.0101	P29291.1	83
	CR_022931.02	Troponin C	6	150	Hom a 6.0101	P29291.1	76
	CR_022930.02	Troponin C	4	130	Hom a 6.0101	P29291.1	86
	CR_022927.02	Troponin C	5	160	Hom a 6.0101	P29291.1	88.67
	CR_009141.02	Troponin C	1	137	Hom a 6.0101	P29291.1	60
	CR_022011.01	Troponin C	3	111	Hom a 6.0101	P29291.1	32.65
	CR_003785.02	Troponin C	4	140	Hom a 6.0101	P29291.1	51.70
	CR_003787.02	Troponin C	5	142	Hom a 6.0101	P29291.1	47.52
	CR_015993.02	Troponin C	6	213	Hom a 6.0101	P29291.1	48.59
7	CR_023727.04	Troponin I	10	217	Pon l 7.0101	P05547.1	84.08
8	CR_005914.02	Triosephosphate isomerase	5	245	Scy p 8.0101	APP94292.1	99.59
	CR_007302.02	Triosephosphate isomerase	4	269	Scy p 8.0101	APP94292.1	60.89
	CR_005730.02	Triosephosphate isomerase	4	269	Scy p 8.0101	APP94292.1	60.89
	CR_006914.02	Triosephosphate isomerase	4	254	Scy p 8.0101	APP94292.1	46.12
9	CR_002704.03	Filamin C	42	2439	Scy p 9.0101	QFI57017.1	98.58
	CR_001162.02	Filamin C	47	2985	Scy p 9.0101	QFI57017.1	26.19
10	CR_008096.02	Hemocyanin	3	676	Pen m 7.0101	AEB77775.1	68.75
	CR_008097.02	Hemocyanin	3	676	Pen m 7.0101	AEB77775.1	69.49
	CR_008098.01	Hemocyanin	3	668	Pen m 7.0101	AEB77775.1	69.55
	CR_008099.02	Hemocyanin	2	677	Pen m 7.0101	AEB77775.1	68.65
	CR_008100.01	Hemocyanin	3	676	Pen m 7.0101	AEB77775.1	68.45
	CR_008101.02	Hemocyanin	3	673	Pen m 7.0101	AEB77775.1	64.78
	CR_021338.01	Hemocyanin	2	664	Pen m 7.0101	AEB77775.1	57.65
	CR_021339.01	Hemocyanin	2	663	Pen m 7.0101	AEB77775.1	58.65
	CR_008519.01	Hemocyanin	2	672	Pen m 7.0101	AEB77775.1	59.25
	CR_001589.01	Hemocyanin	2	634	Pen m 7.0101	AEB77775.1	51.59
	CR_001592.01	Hemocyanin	3	677	Pen m 7.0101	AEB77775.1	51.34
	CR_001594.01	Hemocyanin	3	677	Pen m 7.0101	AEB77775.1	51.19
	CR_001595.01	Hemocyanin	3	678	Pen m 7.0101	AEB77775.1	51.56
	CR_001602.02	Hemocyanin	3	610	Pen m 7.0101	AEB77775.1	52.31
	CR_001602.03	Hemocyanin	3	678	Pen m 7.0101	AEB77775.1	51.86
	CR_025234.03	Hemocyanin	2	518	Pen m 7.0101	AEB77775.1	52.14
	CR_025234.04	Hemocyanin	2	610	Pen m 7.0101	AEB77775.1	52.64
13	CR_001190.02	Fatty acid‐binding protein	4	136	Lit v 13.0101	ADK66280.1	84.56
	CR_010649.02	Fatty acid‐binding protein	4	134	Lit v 13.0101	ADK66280.1	52.27
	CR_022483.02	Fatty acid‐binding protein	4	134	Lit v 13.0101	ADK66280.1	36.36
14	CR_020736.02	Glycogen phosphorylase‐like protein	7	849	Pen m 14.0101	URW11955.1	90.69

^a^ The gene was manually curated based on the sequence similarity search with genomic information in addition to the in silico genome annotation.

Transcriptome analysis revealed that an arginine kinase homolog (group 2) had the highest expression across tissues, followed by tropomyosin (group 1). Muscle proteins including troponin C (group 6), troponin I (group 7), myosin light chain 1 (group 5), and myosin light chain 2 (group 3) were prominently expressed in legs then bodies. Conversely, enzymes like triosephosphate isomerase (group 8) and glycogen phosphorylase‐like protein (group 14) were more expressed in hepatopancreas and eggs. Hemocyanin (group 10) had hepatopancreas‐specific expression. Some myosin light chain 2 homologs similar to calmodulin were more expressed in eggs and hepatopancreas (Figure 1A).

**FIGURE 1:**
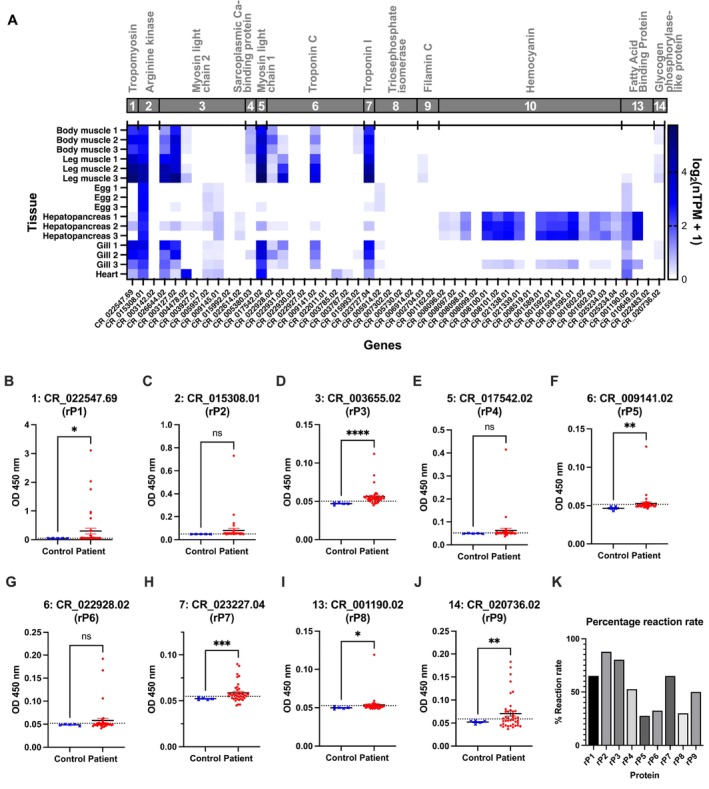
Characterization of putative allergens and allergenicity of recombinant proteins in *Scylla serrata* in crab‐allergic patients from Guangzhou. (A) The gene expression of Decapoda allergen homologs in *S. serrata* is represented by transcripts per million (TPM) normalized by the Gapdh gene, logarithmically transformed, and visualized by a heatmap. Transcriptomes of tissues (except heart) sequenced in triplicates were labeled as 1, 2, and 3 on the *y*‐axis. The *x*‐axis represented the putative allergens from different gene families. (B–J) The ELISA results of the serum samples from the Guangzhou batch revealed the IgE reactivity of each predicted allergen as measured by the absorbance at 450 nm of 40 crab‐positive patients and 5 healthy controls from Guangzhou. The dotted line indicated the cutoff for a positive result calculated from the mean of the controls + 2*standard deviation. Statistically significant results of the difference were displayed with asterisks (*), with * indicating *p* < 0.05, ** indicating *p* < 0.01, *** indicating *p* < 0.001, and **** indicating *p* < 0.0001. (K) Summary of percentages of a positive reaction rate of recombinant proteins.

Nine recombinant proteins were cloned from eight gene families, including two troponin C homologs (Table S5, Figure S5). Forty serum samples from Guangzhou tested positive for *S. serrata* body muscles were used for ELISA (Table S6A, Figure 1B–K). The allergenicity of groups 1, 3, 6, 7, 13, and 14 putative allergens was confirmed with statistical significance (*p* < 0.05) (Figure 1B,D,F,H–J). Positive reaction rates ranged from 27.5% to 85% (Figure 1K). Additionally, 30 serums from Hong Kong crab‐allergic patients tested with the same recombinant proteins further supported the allergenicity of groups 1, 6, and 14 with significance (Table S6B, Figure S6). The only major allergen in both cohorts was tropomyosin. Groups 6 and 14 were potential mid‐tier allergens.

To identify a broader range of potential allergens, immunoblotting using pooled serum from the same 40 Guangzhou patients revealed 10 IgE‐reactive spots against the body muscle protein of the same *S. serrata* (Figure S7). The subsequent mass spectrometry and database search identified five novel allergens, including myosin heavy chain (spots 1&2), enolase (spot 4), actin (spot 5), fructose‐bisphosphate aldolase (spot 6), and glyceraldehyde‐3‐phosphate dehydrogenase (spot 10) (Table S7A). The proteomic identification also mapped seven computationally predicted allergens verifying the in silico prediction (Table S7B).

In conclusion, this study provides the first complete allergen profile of *S. serrata* , identifying 18 allergen gene families, while validating the allergenicity of six allergens. The tissue‐specific expression and comparative genomics allowed the investigation into cross‐reactivities among shellfish allergens, revealing high similarities between the novel and published allergens [6]. These findings will provide insights for component‐resolved diagnostics in managing shellfish allergy.

## Author Contributions

K.F.‐K.A., J.K.W.N., Q.X., and S.K.‐W.T. conceptualized the study. K.F.‐K.A., S.L., and J.K.W.N. performed the experimental work. K.F.‐K.A., C.T.‐Y.L., and W.M. performed the bioinformatics analysis. X.L., C.Y.Y.W., M.F.T., and T.F.L. provided clinical‐related information. S.W.N.A. and X.L. provided recommendations to experimental protocols. K.F.‐K.A. wrote the manuscript. All authors reviewed the manuscript and discussed the results.

## Ethics Statement

The study was approved by the Hospital Ethics Committee of The First Affiliated Hospital of Guangzhou Medical University (Reference number: gyfyy‐2022‐135) for using the patients' sera in immunoassays and immunoblotting with *S. serrata* proteins.

## Conflicts of Interest

The authors declare no conflicts of interest.

## Supporting information


**Figure S1:** all70053‐sup‐0001‐Figures.pdf.
**Figure S2:** all70053‐sup‐0001‐Figures.pdf.
**Figure S3:** all70053‐sup‐0001‐Figures.pdf.
**Figure S4:** all70053‐sup‐0001‐Figures.pdf.
**Figure S5:** all70053‐sup‐0001‐Figures.pdf.
**Figure S6:** all70053‐sup‐0001‐Figures.pdf.
**Figure S7:** all70053‐sup‐0001‐Figures.pdf.

**Table S1:** all70053‐sup‐0002‐Tables.docx.
**Table S2:** all70053‐sup‐0002‐Tables.docx.
**Table S3:** all70053‐sup‐0002‐Tables.docx.
**Table S4:** all70053‐sup‐0002‐Tables.docx.
**Table S5:** all70053‐sup‐0002‐Tables.docx.
**Table S6:** all70053‐sup‐0002‐Tables.docx.
**Table S7:** all70053‐sup‐0002‐Tables.docx.

**Data S1:** all70053‐sup‐0003‐Supinfo1.docx.

## Data Availability

The genome sequencing data related to this study are deposited in NCBI BioProject with the accession PRJNA1191912 and PRJNA1191913.
