# Supplementary material for: Multi‐Omic Analysis of Scylla serrata Reveals the Allergen Landscape of Mud Crabs and Decapoda Species
Source: Allergy. 2025 Sep 9;81(5):1846–50. doi: 10.1111/all.70053 (PMC13139782; doi:10.1111/all.70053)
Supplement: Supplementary file 1 — Figure S1: all70053‐sup‐0001‐Figures.pdf. Figure S2: all70053‐sup‐0001‐Figures.pdf. Figure S3: all70053‐sup‐0001‐Figures.pdf. Figure S4: all70053‐sup‐0001‐Figures.pdf. Figure S5: all70053‐sup‐0001‐Figures.pdf. Figure S6: all70053‐sup‐0001‐Figures.pdf. Figure S7: all70053‐sup‐0001‐Figures.pdf. [file ALL-81-1846-s001.pdf]

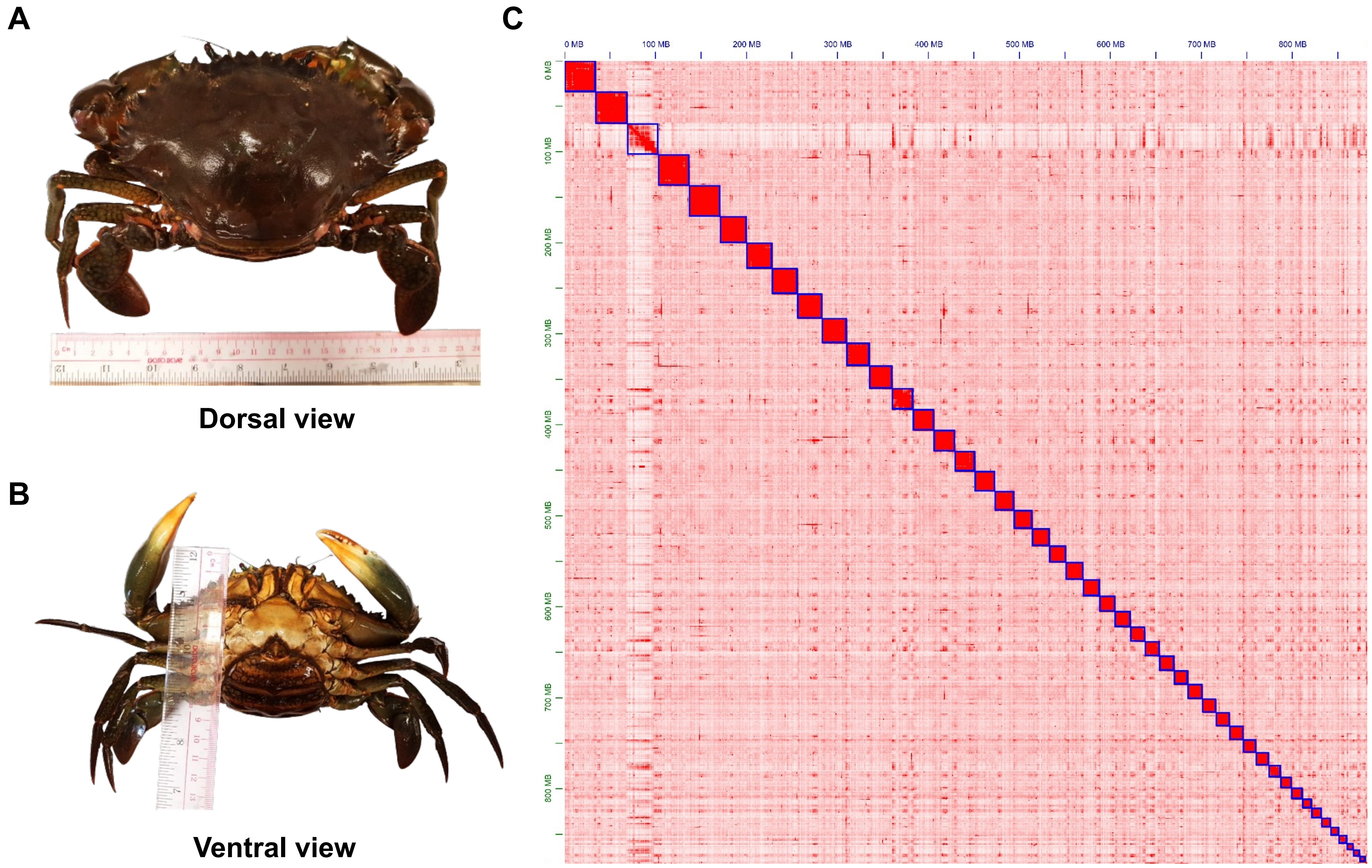

Supplementary Figure S1. Genome construction of female mud crab sample

(A-B) The female mud crab sample dissected for DNA, RNA, and total protein extraction. The female mud crab characterized by a board abdomen (carapace length = 8 cm, width = 15.5 cm) purchased from a local wet market in Hong Kong, sourced from Northern Australia, was used for genomic DNA and RNA extraction. Body muscles, leg muscles, hepatopancreas, gill, and egg were collected and stored in liquid nitrogen. **(A)** and **(B)** represents dorsal and ventral view of the same mud crab, respectively. **(C)** Heatmap for chromosome-level genome construction with the female mud crab. The heatmap in Juicebox represents the interaction frequencies between different genomic regions as determined by Hi-C data. The color intensity of each cell indicates the strength of the interaction, with darker colors representing higher interaction frequencies. 46 pseudo-chromosomes were identified from the heatmap, which were arranged based on their sizes in descending order.

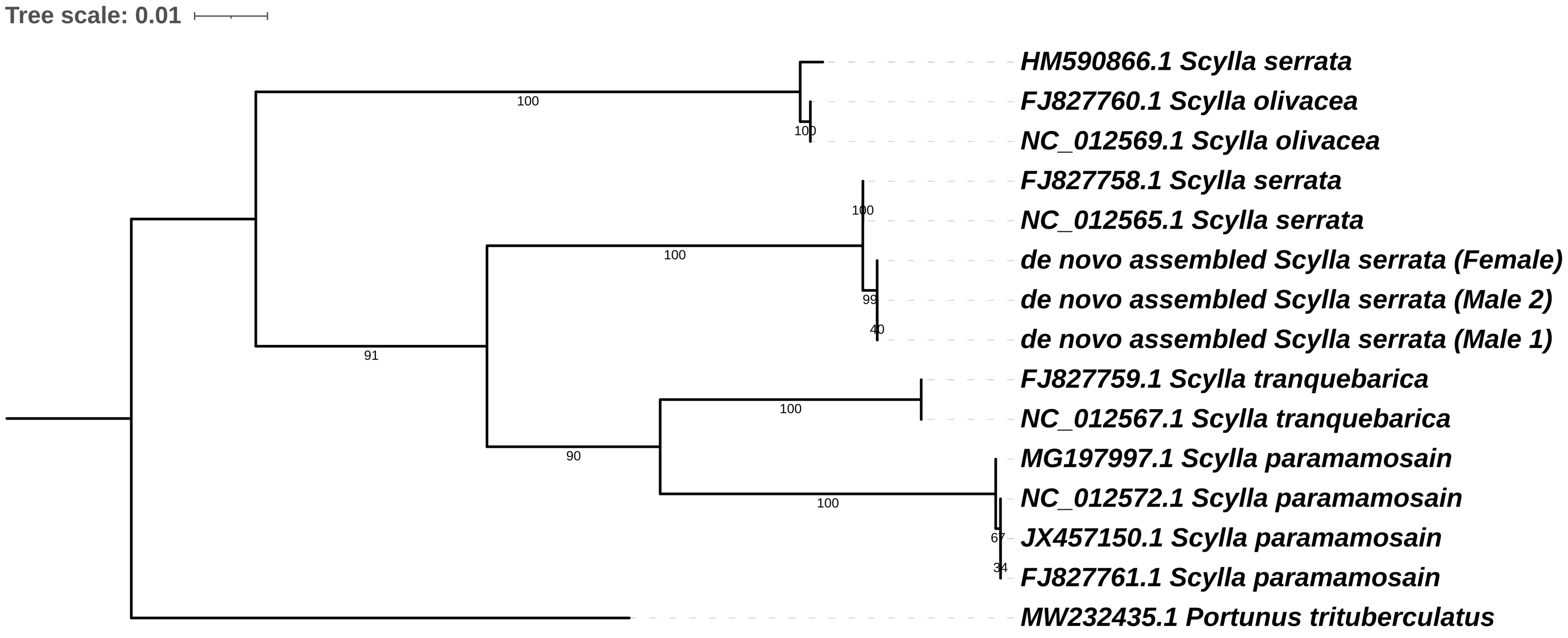

Supplementary Figure S2. Mitochondrial phylogeny of *Scylla* crab species

The COX1 gene sequences from species of *Scylla* genus obtainable in NCBI are used to construct the phylogenetic tree (visualized by iTOL) with the three *de novo* assembled *S. serrata* sequences, including one female species and two male species. The clustering of three de novo assemblies with the *S. serrata* sequence from NCBI confirmed the sequenced species is *S. serrata*.

Supp Figure S2_Ao et al.

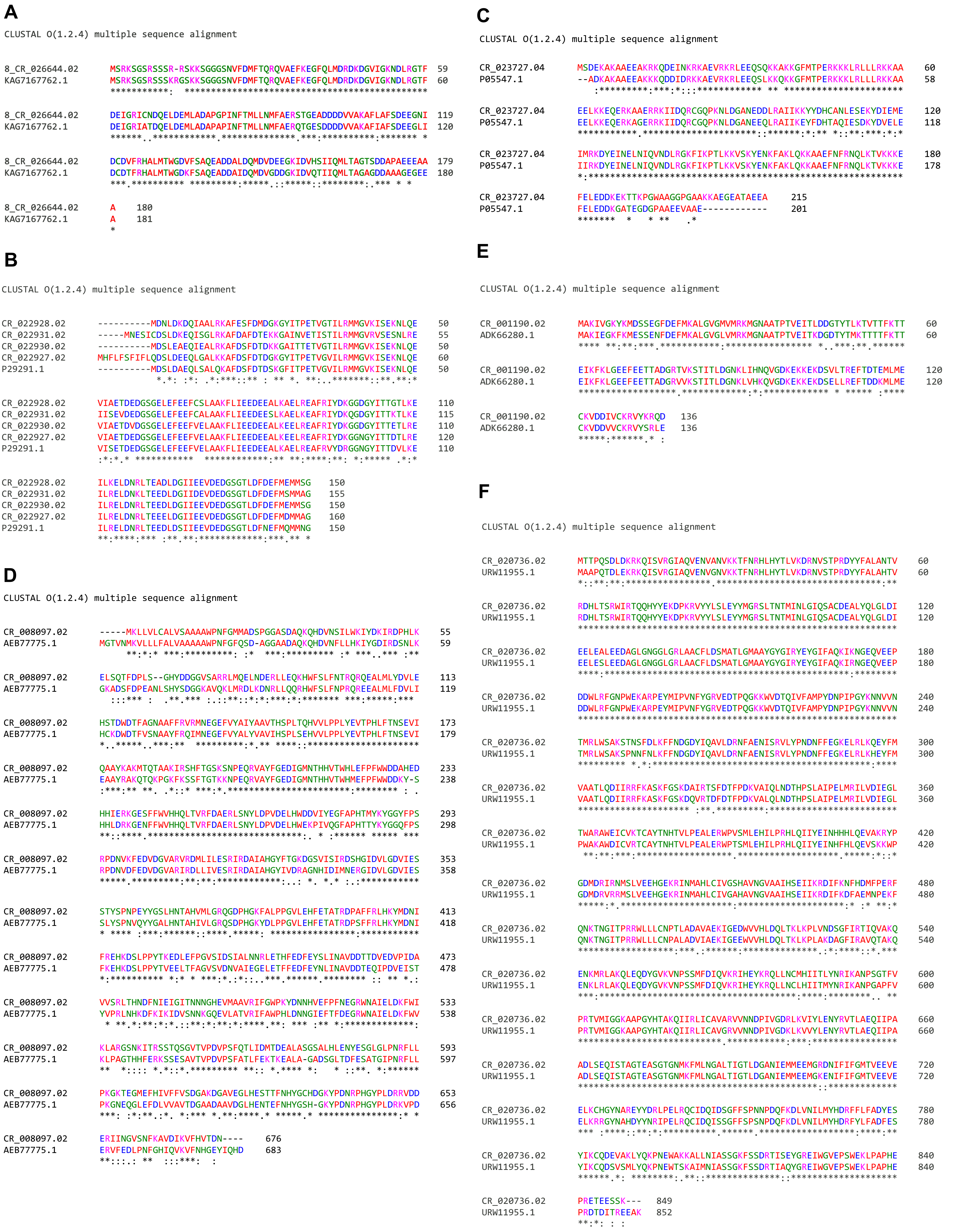

Supplementary Figure S3. Gene family comparison of novel allergens in *S. serrata*

The isoform of curated gene with the most aligned transcriptome reads and the highest sequence homology was used to aligned with the published allergen protein sequence. **(A)** group 3 allergen, myosin light chain 2. **(B)** group 6 allergen, Troponin C with the four tandemly duplicated *S. serrata* genes with similar homology. **(C)** group 7 allergen, Troponin I with the *S. serrata* transcript of the highest coverage. **(D)** group 10 allergen, Hemocyanin. **(E)** group 13 allergen, fatty acid-binding protein. **(F)** group 14 allergen, Glycogen phosphorylase-like protein.

Supp Figure S3_Ao et al.

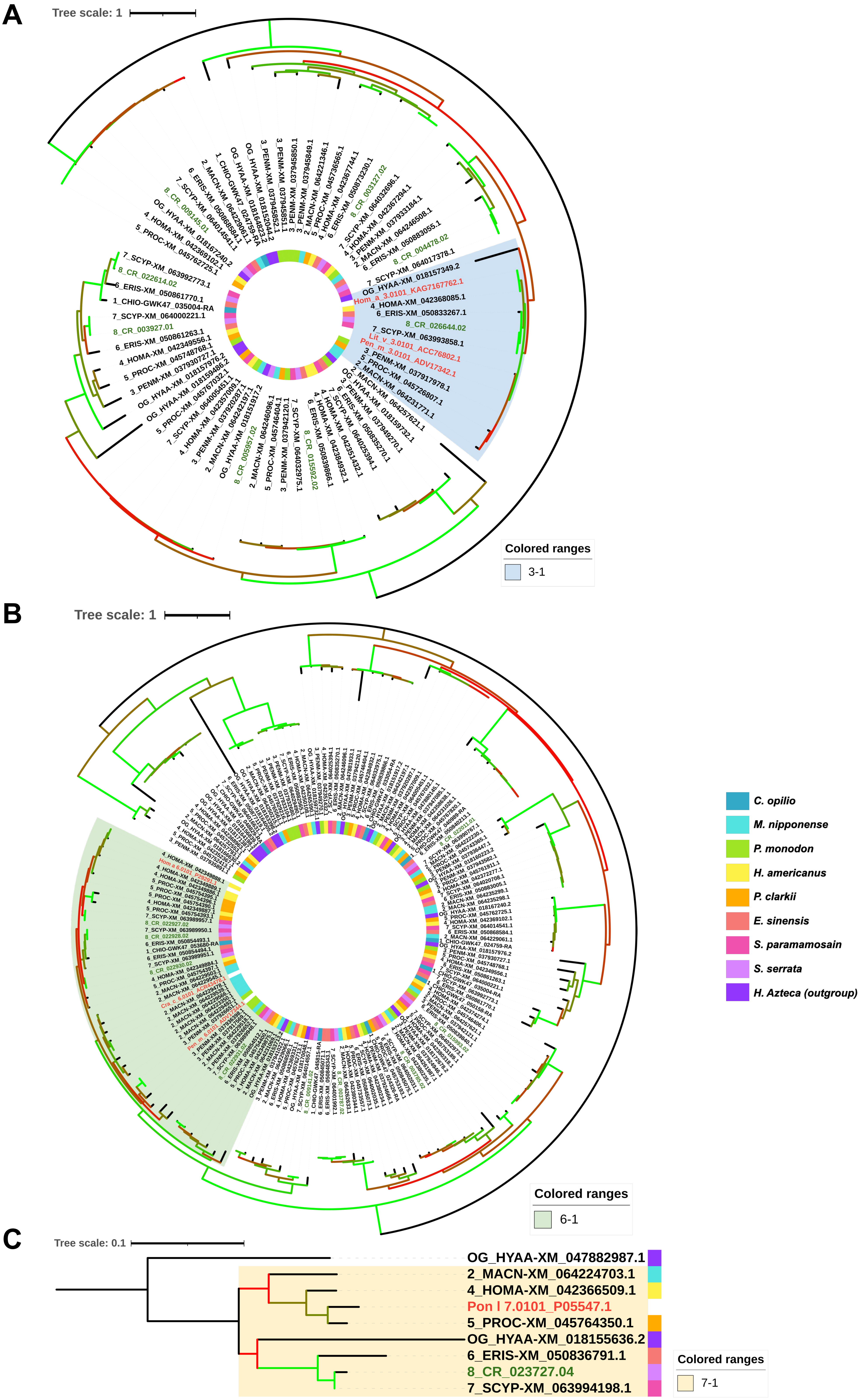

Supplementary Figure S4. Phylogenetic analysis of predicted novel allergens in *S. serrata*

The phylogeny of the novel allergens was elucidated from 8 Decapoda species and an outgroup, and single allergen cluster consisting of *S. serrata* allergen orthologs and published allergens were observed in fivc gene families except for hemocyanin. Bootstrap values were shown by colors, with green color indicating a high bootstrap value. **(A)** Phylogeny of group 3 allergen, myosin light chain 2. **(B)** Phylogeny of group 6 allergen, troponin C. **(C)** Phylogeny of group 7 allergen, troponin I.

Supp Figure S4_Ao et al.

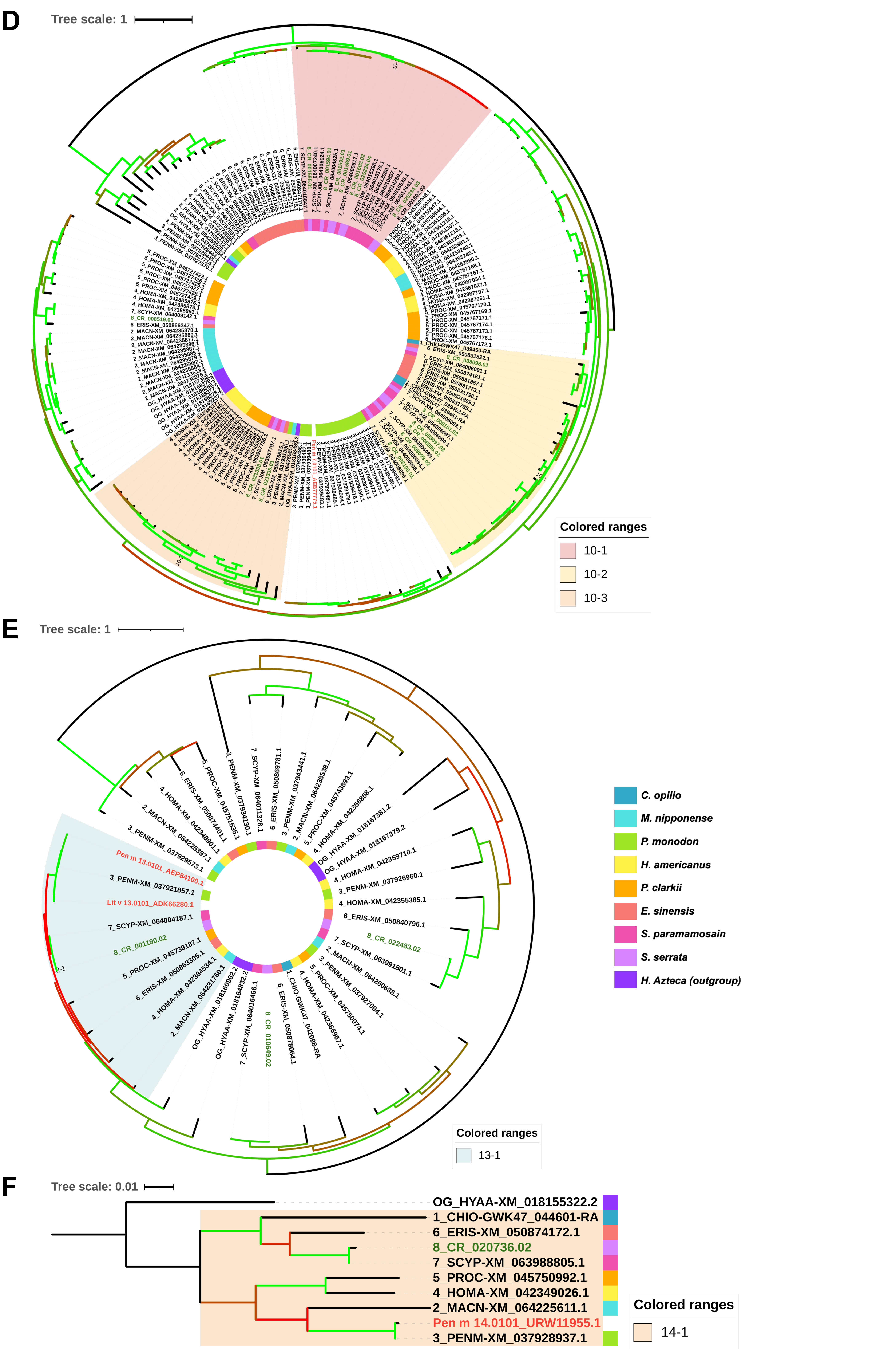

Supplementary Figure S4. Phylogenetic analysis of predicted novel allergens in *S. serrata* (cont'd)

The phylogeny of the novel allergens was elucidated from 8 Decapoda species and an outgroup, and single allergen cluster consisting of *S. serrata* allergen orthologs and published allergens were observed in fivc gene families except for hemocyanin. Bootstrap values were shown by colors, with green color indicating a high bootstrap value. **(D)** Phylogeny of group 10 allergen, hemocyanin. **(E)** Phylogeny of group 7 allergen, troponin I. **(F)** Phylogeny of group 14 allergen, Glycogen phosphorylase-like protein.

Supp Figure S4_Ao et al.

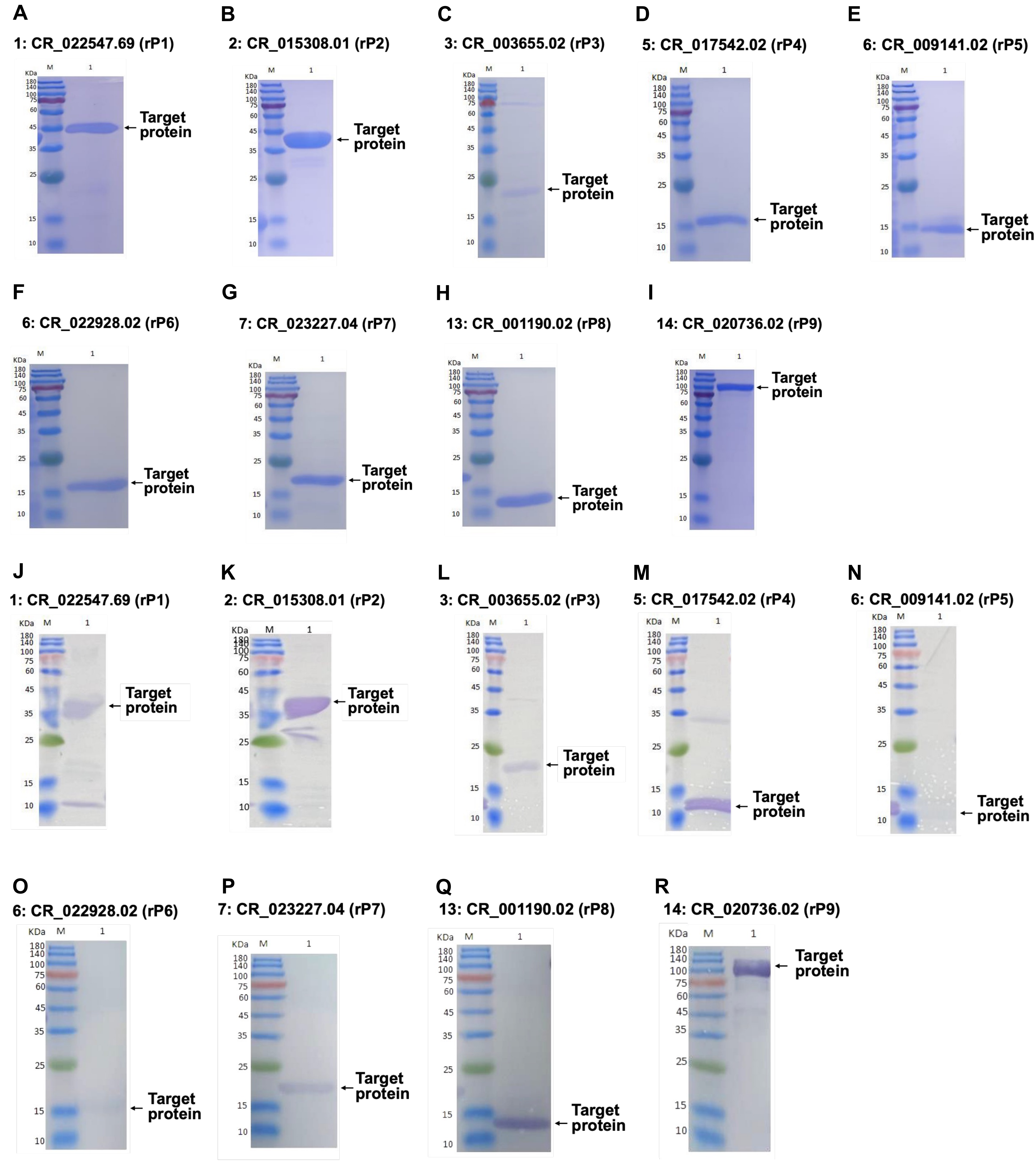

Supplementary Figure S5. Results of molecular cloning of recombinant proteins as demonstrated by SDS-PAGE and Western blotting

(A-I) Each image corresponds to one recombinant protein cloned for immunoassays. Positive cloning results were indicated by SDS-PAGE. In each SDS-PAGE image, the molecular size of each of the nine cloned proteins (1) can be estimated by comparing with the protein marker (M). The fusion protein was successfully purified as a clear band was formed. **(J-R)** Each image corresponds to one recombinant protein cloned for immunoassays. Positive cloning results were indicated by Western blotting. In each Western blotting image, the molecular size of each of the nine cloned proteins (1) can be estimated by the comparsion with the protein marker (M) in the TMB-blotted setting. The fusion protein was successfully purified as a clear band was formed. Image captured by Sangon Biotech (Shanghai, China).

Supp Figure S5_Ao et al.

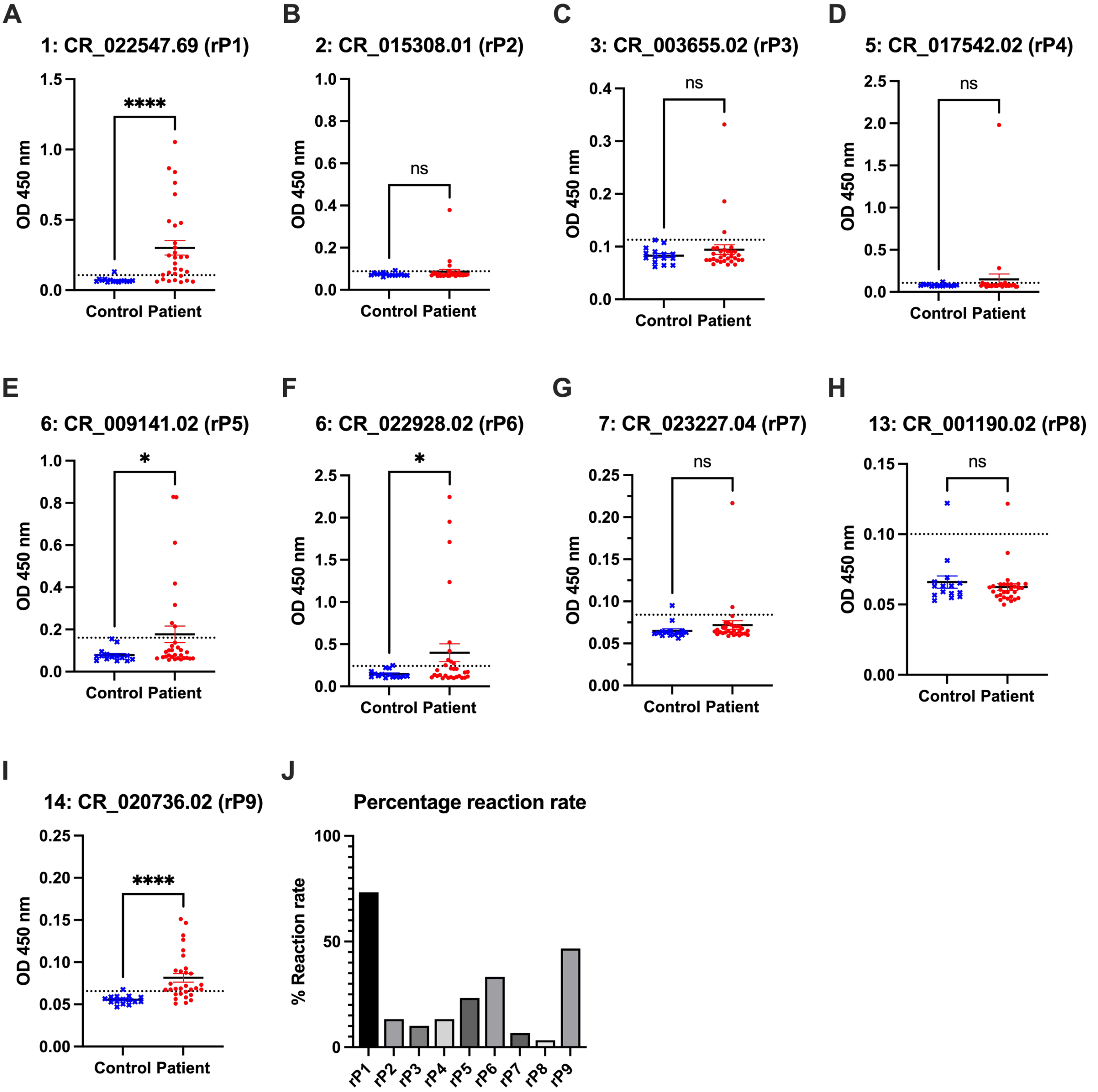

Supplementary Figure S6. Allergenicity of recombinant proteins in *S. serrata* in of crab-allergic patients in Hong Kong

(A-I) The ELISA results of the Hong Kong batch revealed the IgE reactivity of each predicted allergen as measured by the absorbance at 450 nm of 40 crab-positive patients and 5 healthy controls from Hong Kong. The dotted line indicated the cutoff for a positive result calculated from mean of the controls + 2*standard deviation. Statistically significant results (P<0.05) of the difference were displayed with asterisks (*). **(J)** Summary of percentages of a positive reaction rate of recombinant proteins.

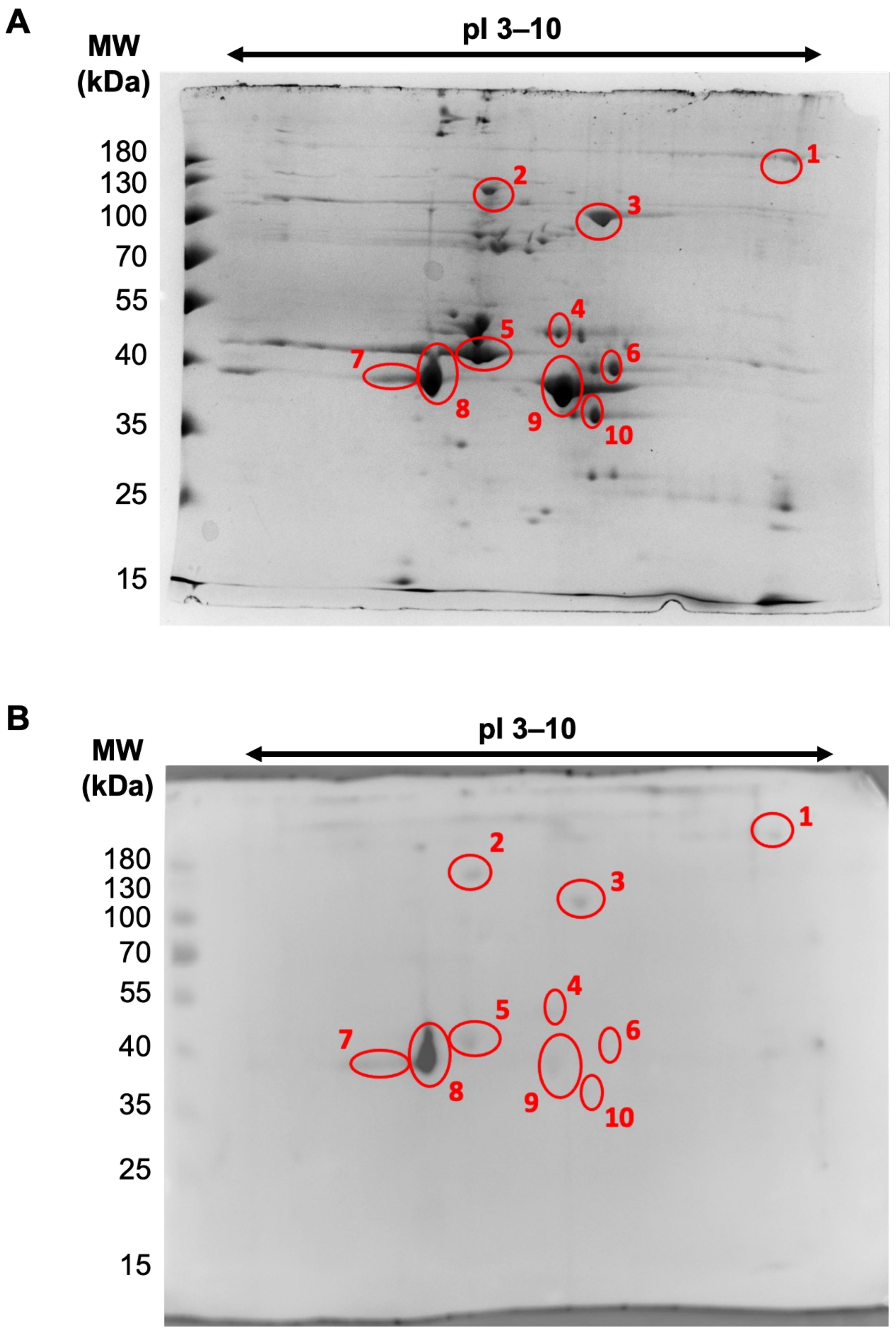

Supplementary Figure S7. 2-D gel electrophoresis and Western blotting

(A) The SDS-PAGE gel separated proteins from *S. serrata* body muscles. The gel image was stained by Coomassie Blue and displayed in greystyle. **(B)** The Western blotting with pooled sera of 40 crab-positive patients in the Guangzhou batch. The immunoblot identified 10 protein spots (numbered 1 to 10) with subsequent identity confirmation by mass spectrometry took place. The 10 proteins were found on the SDS-PAGE correspondingly.
