# Supplementary material for: Multi‐Omic Analysis of Scylla serrata Reveals the Allergen Landscape of Mud Crabs and Decapoda Species
Source: Allergy. 2025 Sep 9;81(5):1846–50. doi: 10.1111/all.70053 (PMC13139782; doi:10.1111/all.70053)
Supplement: Supplementary file 2 — Table S1: all70053‐sup‐0002‐Tables.docx. Table S2: all70053‐sup‐0002‐Tables.docx. Table S3: all70053‐sup‐0002‐Tables.docx. Table S4: all70053‐sup‐0002‐Tables.docx. Table S5: all70053‐sup‐0002‐Tables.docx. Table S6: all70053‐sup‐0002‐Tables.docx. Table S7: all70053‐sup‐0002‐Tables.docx. [file ALL-81-1846-s003.docx]

# Supplementary Table S1. Summary of sequencing runs

## (A) Short read sequencing of male and female crabs

| Lib. | Total base (bp) | Reads number | Read length (bp) | Q20 (%) | Q30 (%) | GC (%) | Sequencing platform | Sequencing library |
| --- | --- | --- | --- | --- | --- | --- | --- | --- |
| Crab-F- stLFR | 173,460,776,200 | 867,303,881*2 | 100 | 95.79 | 87.4 | 41.43 | DNBseq PE100 | stLFR |
| Crab-M1-Illumina | 215,924,546,700 | 719,748,489*2 | 150 | 95.89 | 88.98 | 43 | NEXTseq 2000 PE150 | Illumina |
| Crab-M2-Illumina | 182,259,267,900 | 607,530,893*2 | 150 | 96.07 | 89.4 | 42.5 | NEXTseq 2000 PE150 | Illumina |

## (B) Long read Nanopore sequencing of female crab with ONT library

| **Lib.** | **Total base (bp)** | **Reads number** | **Average read length (bp)** | **Sequencing platform** |
| --- | --- | --- | --- | --- |
| **Crab-ONT** | 34,132,393,062 | 15,921,397 | 2143.8 | GridION Mk1 |

## (C) Hi-C sequencing of female crab with Hi-C library

| **Lib.** | **Reads number** | **Total bases (bp)** | **Read length (bp)** | **Q20_read1 (%)** | **Q20_read2 (%)** | **GC(%)** | **Sequencing platform** |
| --- | --- | --- | --- | --- | --- | --- | --- |
| **Crab-Hi-C** | 314,185,493*2 | 94,255,647,900 | 150 | 98.71 | 96.93 | 41.93 | Illumina NovaSeq 6000 |

# (D) Transcriptome sequencing libraries of female crab

| **Sample/Lib.** | **Tissue** | **Reads number** | **Raw data** | **Read length (bp)** | **Q20(%)** | **Q30(%)** | **GC(%)** | **Sequencing platform** |
| --- | --- | --- | --- | --- | --- | --- | --- | --- |
| **CR_B1** | Body muscle | 52,551,884 | 7,882,782,600 | 150 | 97.8 | 94.06 | 51.39 | NovaSeq 6000 PE150 |
| **CR_B2** | Body muscle | 41,196,094 | 6,179,414,100 | 150 | 96.98 | 91.91 | 50.62 | NovaSeq 6000 PE150 |
| **CR_B3** | Body muscle | 43,211,868 | 6,481,780,200 | 150 | 98.90 | 96.12 | 49.68 | NovaSeq X Plus PE150 |
| **CR_L1** | Leg muscle | 53,237,506 | 7,985,625,900 | 150 | 97.71 | 93.78 | 47.21 | NovaSeq 6000 PE150 |
| **CR_L2** | Leg muscle | 41,929,080 | 6,289,362,000 | 150 | 96.78 | 91.73 | 51.13 | NovaSeq 6000 PE150 |
| **CR_L3** | Leg muscle | 47,276,550 | 7,091,482,500 | 150 | 98.87 | 95.77 | 50.18 | NovaSeq X Plus PE150 |
| **CR_HP1** | Hepatopancreas | 47,281,152 | 7,092,172,800 | 150 | 97.65 | 92.73 | 31.09 | NovaSeq 6000 PE150 |
| **CR_HP2** | Hepatopancreas | 42,648,602 | 6,397,290,300 | 150 | 96.69 | 90.96 | 33.83 | NovaSeq 6000 PE150 |
| **CR_HP3** | Hepatopancreas | 44,646,670 | 6,697,000,500 | 150 | 99.22 | 96.88 | 37.08 | NovaSeq X Plus PE150 |
| **CR_E1** | Egg | 53,467,276 | 8,020,091,400 | 150 | 97.85 | 93.64 | 37.18 | NovaSeq 6000 PE150 |
| **CR_E2** | Egg | 44,355,814 | 6,653,372,100 | 150 | 96.62 | 91.22 | 46.94 | NovaSeq 6000 PE150 |
| **CR_E3** | Egg | 41,873,294 | 6,280,994,100 | 150 | 99.09 | 96.49 | 46.87 | NovaSeq X Plus PE150 |
| **CR_G1** | Gill | 41,395,806 | 6,209,370,900 | 150 | 96.96 | 91.58 | 41.14 | NovaSeq 6000 PE150 |
| **CR_G2** | Gill | 42,030,210 | 6,304,531,500 | 150 | 96.77 | 91.17 | 36.97 | NovaSeq 6000 PE150 |
| **CR_G3** | Gill | 44,705,480 | 6,705,822,000 | 150 | 99.25 | 96.99 | 41.07 | NovaSeq X Plus PE150 |
| **CR_H** | Heart | 55,811,482 | 8,371,722,300 | 150 | 97.73 | 93.82 | 52.4 | NovaSeq 6000 PE150 |

# Supplementary Table S2. Statistics of the three draft genomes of *S. serrata*

**(A) Contiguity assessment by QUAST**

|  | Female | Male 1 | Male 2 |
| --- | --- | --- | --- |
| Assembly size (with N) (bp) | 927,944,506 | 901,836,538 | 940,646,025 |
| Assembly size (without N) | 879,052,681 | 731,608,544 | 714,641,884 |
| GC content (%) | 41.20% | 41.43% | 41.44 |
| Scaffold number | 472,644 | 592,217 | 666,081 |
| Scaffold N50 (bp) | 4,752 | 8,336 | 8,910 |
| Longest scaffold (bp) | 310,262 | 105,757 | 98,440 |
| N's per 100 kbp | 5,031 | 5,632.71 | 7,915.26 |
| Contig number | 616,222 | 1,011,490 | 1,318,823 |
| Contig N50 (bp) | 2,863 | 6,259 | 3,523 |
| Longest contig (bp) | 60,596 | 83,364 | 48,676 |

**(B) Completeness assessment by BUSCO**

| Types of BUSCOs | Female | Male 1 | Male 2 |
| --- | --- | --- | --- |
| Complete BUSCOs (C) | 66.6% | 72.2% | 69.5% |
| • Complete and Single-copy BUSCOs (S) | 56.7% | 71.8% | 69.1% |
| • Complete and duplicated BUSCOs (D) | 9.9% | 0.4% | 0.4% |
| Fragmented BUSCOs (F) | 22.7% | 19.9% | 20.1% |
| Missing BUSCOs (M) | 10.7% | 7.9% | 10.4% |
| Total BUSCO groups searched | **1,013** | **1,013** | **1,013** |

BUSCO Lineage Dataset used: Arthropoda (arthropoda_odb10.2020-09-10)

# Supplementary Table S3. Statistics of the chromosome-level genome of *S. serrata*

## (A) Comparison between long read polished and Hi-C scaffolded *S. serrata* genome assemblies

|  | **stLFR**  (draft genome)* | **stLFR & ONT**  (scaffold-level assembly) | **stLFR, ONT & Hi-C**  (chromosome-level assembly, all scaffolds) | **Chromosome assembly** (chromosome-level assembly, anchored chromosomes) |
| --- | --- | --- | --- | --- |
| **Assembly size (with N) (bp)** | 927,944,506 | 951,777,564 | 952,710,116 | 882,908,311 |
| **Assembly size (without N)** | 879,052,681 | 932,431,703 | 931,991,447 | 868,846,239 |
| **GC content (%)** | 41.20% | 41.21% | 41.21% | 41.35% |
| **Pseudo-chromosome number** | N.A. | N.A. | 46 | 46 |
| **Scaffold number** | 472,644 | 6,812 | 6,132 | 46 |
| **Scaffold N50 (bp)** | 4,752 | 526,398 | 21,436,738 | 21,975,617 |
| **Longest scaffold (bp)** | 310,262 | 3,478,985 | 34,991,454 | 34,991,454 |
| **N's per 100 kbp** | 5,031 | 2,033 | 2,173 | 1,593 |
| **Contig number** | 616,222 | 20,263 | 23,262 | 12,086 |
| **Contig N50 (bp)** | 2,863 | 310,669 | 287,685 | 317,722 |
| **Longest contig (bp)** | 60,596 | 2,839,753 | 2,726,140 | 2,726,140 |

***** Statistics for the initial Draft Genome (first data column) are also presented in Table S2A for direct comparison with the male assemblies.

## (B) Genome completeness assessment of chromosome-level genome assembly (all scaffolds) by BUSCO

|  | **Lineage Dataset (odb10)** | | |
| --- | --- | --- | --- |
| **Types of BUSCOs** | **Arthropoda** | **Eukaryota** | **Metazoa** |
| **Complete BUSCOs (C)** | 92.20% | 94.10% | 90.40% |
| **• Complete and Single-copy BUSCOs (S)** | 91.50% | 93.70% | 89.80% |
| **• Complete and duplicated BUSCOs (D)** | 0.70% | 0.40% | 0.60% |
| **Fragmented BUSCOs (F)** | 5.20% | 4.30% | 6.30% |
| **Missing BUSCOs (M)** | 2.60% | 1.60% | 3.30% |
| **Total BUSCO groups searched** | **1,013** | **255** | **954** |

BUSCO Lineage Dataset used: Arthropoda (arthropoda_odb10.2020-09-10), Eukaryota (eukaryota_odb10.2020-09-10), Metazoa (metazoa_odb10.2021-02-24)

## (C) Repeat and sequence annotation results of the *S. serrata* genome

| **Repeat contents** | 41.50% |
| --- | --- |
| **Protein-coding gene count** | 26,643 |
| **• Functional genes predicted by BLAST** | 13,391 |
| **• InterPro Domains** | 7,897 |
| **• GO Terms** | 13,203 |
| **tRNA gene count** | 2,532 |
| **rRNA gene count** | 206 |
| **Total annotated gene count** | 29,381 |
| **Complete and Single-copy BUSCOs (Dataset: arthropoda_odb10)** | 93.00% |

# Supplementary Table S4. BLAST identification of *S. serrata* putative allergens

## (A) Known allergens in *S. paramamosain*

| **Group** | **Allergen** | **GenBank ID** | **Biochemical name** | **Top matched gene ID** | **Top matched scaffold** | **Partial sequence identity** |
| --- | --- | --- | --- | --- | --- | --- |
| **Group 1** | Scy p 1 | ABS12233.1 | Tropomyosin | CR_022547.01 | HiC_scaffold_40 | 100% |
| **Group 2** | Scy p 2 | AFA45340.1 | Arginine kinase | CR_015308.01 | HiC_scaffold_23 | 99.70% |
| **Group 4** | Scy p 4 | AFJ80778.1 | Sarcoplasmic Ca+ binding protein | CR_005380.01 | HiC_scaffold_6 | 98.30% |
| **Group 5*** | Scy p 3 | QDH76468.1 | Myosin light chain 1 | CR_017542.01 | HiC_scaffold_28 | 100% |
| **Group 8** | Scy p 8 | APP94292.1 | Triosephosphate isomerase | CR_005914.01 | HiC_scaffold_7 | 100% |
| **Group 9** | Scy p 9 | QFI57017.1 | Filamin C | CR_002705.01 | HiC_scaffold_3 | 100% |

* Scy p 3 (QDH76468.1) has a BLASTP result mapped to the Myosin light chain 1, suggesting its grouping as a group 5 allergen to be consistent across Decapoda species.

## (B) Novel allergens in the Decapoda order

| **Group** | **Allergen** | **GenBank ID** | **Species name** | **Common Name** | **Biochemical name** | **Top matched gene ID** | **Top matched scaffold** | **Partial sequence identity** |
| --- | --- | --- | --- | --- | --- | --- | --- | --- |
| **Allergens in Decapoda of Animalia Arthropoda** | | | | | | | | |
| **Group 3** | **Hom a 3** | KAG7167762.1 | *Homarus americanus* | American lobster | Myosin light chain 2 | CR_003127.01 | HiC_scaffold_5248 | 90.20% |
| **Group 6** | **Cra c 6** | ACR43478.1 | *Crangon crangon* | North Sea shrimp | Troponin C | CR_022928.01 | HiC_scaffold_42 | 81.80% |
| **Group 7** | **Pon l 7** | P05547.1 | *Pontastacus leptodactylus* | Narrow-clawed crayfish | Troponin I | CR_023727.01 | HiC_scaffold_44 | 100% |
| **Group 10*** | **Pen m 7** | AEB77775.1 | *Penaeus monodon* | Black tiger shrimp | Hemocyanin | CR_008097.01 | HiC_scaffold_10 | 71.90% |
| **Group 13** | **Pen m 13** | ADK66280.1 | *Litopenaeus vannamei* | White shrimp | Fatty acid binding protein | CR_001190.01 | HiC_scaffold_2 | 87.90% |
| **Group 14** | **Pen m 14** | OM156460.1 | *Penaeus monodon* | Black tiger shrimp | Glycogen phosphorylase-like protein | CR_020736.01 | HiC_scaffold_36 | 97.80% |
| **Allergens in Animalia Mollusca** | | | | | | | | |
| **Group 15** | **Rap v 2** | QPB41107.1 | *Rapana venosa* | Veined rapa whelk | Paramyosin | CR_007759.01 | HiC_scaffold_8 | 46.70% |

* Hemocyanin was identified as group 7 allergen in *Penaeus* (*P.*) *monodon*. However, troponin I was identified as group 7 allergen in *Pontastacus* (*P.*) *leptodactylus* at an earlier time. As such, Hemocyanin has been reclassified as a group 10 allergen.

**Supplementary Table S5. List of recombinant proteins cloned for immunoassays**

A list of successfully cloned recombinant proteins for ELISA testing with allergic patients’ samples from Guangzhou and Hong Kong.

| Recombinant Protein ID | **Hi-C gene locus** | **Allergen group** | **Successful cloning** |
| --- | --- | --- | --- |
| rP1 | CR_022547.69 | Group 1 (Tropomyosin) | Yes |
| rP2 | CR_015308.01 | Group 2 (Arginine kinase) | Yes |
| rP3 | CR_003127.02 | Group 3 (Myosin light chain 2) | Yes |
| rP4 | CR_017542.02 | Group 5 (Myosin light chain 1) | Yes |
| rP5 | CR_009141.02 | Group 6 (Troponin C) | Yes |
| rP6 | CR_022928.02 | Group 6 (Troponin C) | Yes |
| rP7 | CR_023227.04 | Group 7 (Troponin I) | Yes |
| rP8 | CR_001190.02 | Group 13 (Fatty Acid Binding Protein) | Yes |
| rP9 | CR_020736.02 | Group 14 (Glycogen phosphorylase-like protein) | Yes |

# Supplementary Table S6. Metadata of crab-allergic patients selected for immunoassays

**(A) Guangzhou batch**

Serum samples were collected from patients with a clinical diagnosis of an allergic disorder or a positive test for crab-specific IgE (ImmunoCAP class 1 or higher).

| **#** | **Sample ID** | **Sex** | **Age (y)** | **ImmunoCAP, f23 (kUA/L)** | **Allergy class** | **ELISA (OD 450)** | **Diagnosis** |
| --- | --- | --- | --- | --- | --- | --- | --- |
| 1 | 23454 | F | 52 | 1.83 | 2 | 0.194 | N.A. |
| 2 | 25419 | M | 48 | 2.63 | 2 | 0.21 | N.A. |
| 3 | 28143 | F | 17 | 4.91 | 3 | 0.172 | N.A. |
| 4 | 29998 | F | 24 | 1.92 | 2 | 0.307 | N.A. |
| 5 | 31560 | F | 28 | 0.74 | 2 | 0.13 | chest distress |
| 6 | 32856 | F | 17 | 2.75 | 2 | 0.259 | N.A. |
| 7 | 33615 | F | 26 | 1.16 | 2 | 0.3 | Conjunctivitis |
| 8 | 85083 | M | 32 | 3 | 2 | 0.935 | allergic rhinitis |
| 9 | 85100 | M | 31 | 6.3 | 3 | 0.403 | chronic urticaria |
| 10 | 85160 | M | 52 | 4.08 | 3 | 0.139 | itching |
| 11 | 85241 | F | 30 | 1.16 | 2 | 0.13 | urticaria |
| 12 | 85378 | M | 68 | 1.17 | 2 | 0.4 | urticaria |
| 13 | 86633 | M | 26 | 1.18 | 2 | 0.944 | N.A. |
| 14 | 86719 | N.A. | N.A. | 1.16 | 2 | 0.151 | N.A. |
| 15 | 86901 | F | 33 | 0.92 | 2 | 0.183 | N.A. |
| 16 | 87190 | F | 51 | 1.42 | 2 | 0.153 | acute urticaria |
| 17 | 90170 | F | 27 | 1.07 | 2 | 0.177 | acute urticaria |
| 18 | 90522 | F | 34 | 1.27 | 2 | 0.133 | chronic urticaria |
| 19 | 90786 | M | 8 | 3.43 | 2 | 0.16 | allergic rhinitis |
| 20 | 91472 | M | 18 | 1.24 | 2 | 0.149 | itching |
| 21 | 91571 | F | 52 | 1.28 | 2 | 0.134 | allergic dermatitis |
| 22 | 92282 | F | 34 | 8.27 | 3 | 0.142 | N.A. |
| 23 | 92315 | M | 31 | 5.52 | 3 | 0.496 | atopic dermatitis |
| 24 | 92388 | M | 35 | 4.7 | 3 | 0.762 | N.A. |
| 25 | 92530 | F | 18 | 2.67 | 2 | 0.138 | urticaria |
| 26 | 92875 | M | 6 | 10.5 | 3 | 0.153 | N.A. |
| 27 | 95465 | M | 11 | 6.92 | 3 | 0.131 | N.A. |
| 28 | 97455 | M | 45 | 14.2 | 3 | 0.197 | allergic dermatitis |
| 29 | H8669 | M | 5 | N.A. | N.A. | 0.146 | cough |
| 30 | H8718 | M | 9 | N.A. | N.A. | 0.145 | bronchial asthma |
| 31 | H8787 | F | 55 | N.A. | N.A. | 0.288 | bronchial asthma |
| 32 | H8848 | M | 48 | N.A. | N.A. | 0.156 | bronchial asthma |
| 33 | H8940 | F | 15 | N.A. | N.A. | 0.174 | bronchial asthma |
| 34 | H9116 | M | 5 | N.A. | N.A. | 0.45 | wheezing |
| 35 | H9170 | M | 36 | N.A. | N.A. | 0.416 | bronchial asthma |
| 36 | H9195 | F | 7 | N.A. | N.A. | 0.222 | bronchial asthma |
| 37 | X7790 | F | 24 | 6.1 | 3 | 0.46 | cough |
| 38 | X7859 | F | 3 | 1.68 | 2 | 0.14 | atopic dermatitis |
| 39 | X7870 | M | 5 | 0.47 | 1 | 0.346 | atopic dermatitis |
| 40 | X7878 | F | 1 | N.A. | N.A. | 0.465 | allergic sleeping disorder |

* N.A. refers to the data which was not available from the hospital record.

**(B) Hong Kong batch**

Serum samples were collected from patients with a clinical diagnosis of an allergic disorder (e.g., allergic rhinitis, asthma, or atopic dermatitis) or a history of food allergy symptoms after ingestion of shellfish.

| # | Sample ID | Sex | Age (y) | ImmunoCAP, f23 (kUA/L) | Allergy class |
| --- | --- | --- | --- | --- | --- |
| 1 | SA0640 | M | 3 | 59.8 | 5 |
| 2 | SA0230 | M | 20 | 57.2 | 5 |
| 3 | SA0410 | M | 17 | 42.3 | 4 |
| 4 | SA0482 | M | 14 | 38.3 | 4 |
| 5 | SA0540 | F | 6 | 37.4 | 4 |
| 6 | SA0314 | M | 2 | 27.6 | 4 |
| 7 | SA0184 | M | 1 | 24.9 | 4 |
| 8 | SA0657 | F | 5 | 23.1 | 4 |
| 9 | SA0477 | M | 18 | 14.6 | 3 |
| 10 | SA0652 | M | 31 | 14.6 | 3 |
| 11 | SA0353 | F | 8 | 14.5 | 3 |
| 12 | SA0407 | M | 5 | 14.3 | 3 |
| 13 | SA0409 | F | 20 | 8.74 | 3 |
| 14 | SA0508 | M | 21 | 8.48 | 3 |
| 15 | SA0472 | M | 43 | 7.72 | 3 |
| 16 | SA0119 | M | 7 | 7.6 | 3 |
| 17 | SA0567 | F | 19 | 7.04 | 3 |
| 18 | SA0496 | F | 11 | 6.8 | 3 |
| 19 | SA0635 | F | 54 | 6.09 | 3 |
| 20 | SA0481 | M | 5 | 5.9 | 3 |
| 21 | SA0647 | M | 31 | 5.88 | 3 |
| 22 | SA0345 | M | 9 | 5.74 | 3 |
| 23 | SA0624 | F | 28 | 5.4 | 3 |
| 24 | SA0654 | F | 55 | 5.31 | 3 |
| 25 | SA0329 | F | 3 | 4.94 | 3 |
| 26 | SA0533 | M | 22 | 4.92 | 3 |
| 27 | SA0656 | M | 2 | 4.5 | 3 |
| 28 | SA0489 | F | 27 | 4.09 | 3 |
| 29 | SA0480 | M | 14 | 3.97 | 3 |
| 30 | SA0486 | M | 8 | 3.74 | 3 |

# Supplementary Table S7. Results of mass spectrometry

## (A) Best-matched proteins identified from the immunoblotting.

## The best-matched protein with the highest score in the database search from each protein spot from the gel was summarized.

| **Protein spot #** | **Gene ID** | **Biological function (WHO/IUIS allergen ID)** | **Score** | **Mass (Da)** | **pI** | **# matches** | **# significant peptide matches** | **Coverage** |
| --- | --- | --- | --- | --- | --- | --- | --- | --- |
| **1** | CR_006567.01 | Myosin heavy chain, muscle | 2,821 | 219,685 | 5.87 | 138 | 105 | 46.9 |
| **2** | CR_007759.01 | Myosin heavy chain, muscle | 4,399 | 255,738 | 5.93 | 209 | 157 | 32.9 |
| **3** | CR_002705.01 | Filamin C (Scy p 9.0101) | 5,666 | 99,851 | 6.79 | 304 | 218 | 67.3 |
| **4** | CR_007687.01 | Enolase | 1,891 | 47,616 | 6.12 | 103 | 80 | 71.7 |
| **5** | CR_019306.01 | Actin-3, muscle-specific | 2,336 | 42,098 | 5.24 | 114 | 88 | 59.6 |
| **6** | CR_004773.01 | Fructose-bisphosphate aldolase | 3,631 | 40,287 | 6.62 | 141 | 114 | 84.1 |
| **7** | CR_015308.01 | Arginine kinase (Scy p 2.0101) | 834 | 40,617 | 6.18 | 40 | 33 | 66.9 |
| **8** | CR_022547.01 | Tropomyosin (Scy p 1.0101) | 2,227 | 25,357 | 4.58 | 114 | 86 | 50 |
| **9** | CR_015308.01 | Arginine kinase (Scy p 2.0101) | 7,176 | 40,617 | 6.18 | 346 | 266 | 83.2 |
| **10** | CR_005735.01 | Glyceraldehyde-3-phosphate dehydrogenase | 2,461 | 36,029 | 6.6 | 133 | 99 | 82.3 |

## (B) Shared matches in the putative allergens between the *in silico* prediction and the proteomic data

The results of the *in silico* allergen prediction was verified from the mass spectrometry by searching for shared peptide sequences from the putative allergens which also present in the database search from mass spectrometry.

| **Allergen group** | **Curated protein ID** | **Biochemical function** | **MS matched protein** | **# significant protein spots*** | **Significant protein spot #** |
| --- | --- | --- | --- | --- | --- |
| 1 | CR_022547.69 | Tropomyosin | CR_022547.01 | 4 | 7,8,9,10 |
| 2 | CR_015308.01 | Arginine kinase | CR_015308.01 | 9 | 2,3,4,5,6,7,8,9,10 |
| 3 | CR_005957.02 | Myosin light chain 2 | CR_005957.01 | 3 | 1,5,7 |
| 7 | CR_023727.04 | Troponin I | CR_023727.01 | 1 | 5 |
| 9 | CR_002704.03 | Filamin C | CR_002705.01 | 7 | 3,4,5,6,7,8,10 |
| 10 | CR_008096.02 | Hemocyanin | CR_008096.01 | 1 | 2 |
| 10 | CR_008098.01 | Hemocyanin | CR_008098.01 | 1 | 2 |
| 10 | CR_008100.01 | Hemocyanin | CR_008100.01 | 2 | 2,5 |
| 10 | CR_008101.02 | Hemocyanin | CR_008101.01 | 1 | 2 |
| 10 | CR_021338.01 | Hemocyanin | CR_021338.01 | 1 | 3 |
| 14 | CR_020736.02 | Glycogen phosphorylase-like protein | CR_020736.01 | 1 | 3 |

* Significant protein spots refer to the spots with at least one significant peptide (p-value < 0.05) when performing the database search with the MS result.
