# Supplementary material for: Multi‐Omic Analysis of Scylla serrata Reveals the Allergen Landscape of Mud Crabs and Decapoda Species
Source: Allergy. 2025 Sep 9;81(5):1846–50. doi: 10.1111/all.70053 (PMC13139782; doi:10.1111/all.70053)
Supplement: Supplementary file 3 — Data S1: all70053‐sup‐0003‐Supinfo1.docx. [file ALL-81-1846-s002.docx]

**Supplementary methods: Multi-omic analysis of *Scylla serrata* reveals the allergen landscape of mud crabs and Decapoda species**

**Study design:** The study adopted a multi-omic approach to identify and characterize the allergen landscape of *S. serrata*. By combining genomic and transcriptomic data, it allows a proteome to be annotated *in silico* for predicting allergens and their gene expressions, which has demonstrated the utility of bioinformatic tools in characterizing individual allergens ^1, 2^.

## 1. Genomic DNA and RNA extraction

A female mud crab (Figure S1A, S1B) and two male mud crabs were purchased from a local wet market in Hong Kong. The mud crabs were sourced from wild water from the coast in Northern Australia, which was one of the commonly found habitats of *S. serrata*. The selection enabled the representation of the mud crabs that would commonly appear in the diet of people in Hong Kong and East Asia, which also matched to the common habitats of *S. serrata*. Although the use of wild samples could avoid the issue of artificial selection; however, some growth conditions, including microclimate and their food source, may not be well-controlled.

Six tissues were dissected from the mud crabs: body muscles, leg muscles, hepatopancreas, eggs, gills, and heart. The extraction of genomic DNA (gDNA) began with the tissue homogenization of the body muscles with liquid nitrogen. Following the manufacturer’s guidelines, gDNA was extracted from the homogenized sample using the Puregene Tissue Kit (Qiagen, Germany) and subsequently purified using the Wizard Genomic DNA Purification Kit (Promega, USA). Additionally, RNA was extracted from the six tissues using TRIzol reagent and the PureLink RNA Mini Kit (Thermo Fisher Scientific, USA), following the manufacturer’s instructions.

## 2. Genome sequencing, assembly, and improvement

To construct the reference genome of *S. serrata* using the female sample, hybrid genome sequencing was performed combining short reads using DNBseq with the single-tube long fragment reads (stLFR) library (Table S1A), and long reads using Oxford Nanopore Technologies (ONT) GridION with the library prepared by the Ligation Sequencing Kit (SQK-LSK110) (Table S1B). A draft genome were constructed by stLFR reads alone. An initial contig-level genome was assembled from the ONT reads using the Flye (v2.9) ^3^. Polishing steps employed Racon (v1.4.3) ^4^ for long reads and Pilon (v1.24) ^5^ for short reads. Scaffolding and gap filling were performed by SSPACE-LongRead (v1.1) ^6^ and TGS-GapCloser (v1.2.1) ^7^ respectively, resulting in a scaffold-level genome. The genome quality was assessed by QUAST (v5.2.0) ^8^ for genome statistics, and BUSCO (v5.4.6) ^9^ for completeness assessment.

Further improvement of the genome to the chromosome level has taken place by the Hi-C technique. The Hi-C library was constructed with the DpnII as the restriction enzyme for cleavage, which was subsequently sequenced on the NovaSeq 6000 with the Hi-C library (Table S1C). The raw reads were used to scaffold the scaffold-level S. serrata genome with Juicer (v1.6) ^10^ for contact map generation, 3D-DNA (v180922) ^11^ for scaffolding with Hi-C data, and Juicebox (v2.15) ^12^ for visualizing the matrix and pseudo-chromosomes.

Additionally, draft genomes for the two male samples were assembled. Genomic DNA was sequenced using short reads with libraries prepared by the Illumina DNA Prep Kit (Table S1A). The raw reads were assembled into draft genomes using SOAPdenovo2 ^13^. The quality of these draft assemblies was similarly assessed using QUAST and BUSCO. The resulting genomes from two males, together with the genome assembled from the female sample, were used to construct the mitochondrial phylogeny with other species of the *Scylla* genus with mitochondrial sequences available on the NCBI for species validation.

## 3. Transcriptome sequencing and assembly

Transcriptome sequencing was performed for the six extracted tissues with the same female *S. serrata* sample used in genome sequencing with short read sequencing (Table S1D). Except for heart tissue, transcriptomes for five other tissues (body muscles, leg muscles, hepatopancreas, eggs, and gills) are sequenced in triplicates. To construct a comprehensive transcript set for *S. serrata*, a dual-approach strategy combining both reference-based and *de novo* assembly was employed. First, the RNAseq reads were aligned to our *de novo* genome assembly by Hisat2 (v2.2.1) ^14^. Subsequently, StringTie (v2.2.1) ^15^ and Gffread (v0.12.7) ^16^ were used to assemble these mapped reads into transcripts, for defining exon-intron boundaries based on the genomic scaffold. In parallel, *de novo* assembly of the transcriptome was done by Trinity (v2.15.1) ^17^ to capture novel transcripts or transcripts from unannotated genomic regions. Finally, these two sets of transcripts were merged to create a final transcript set that was used as evidence for the downstream genome annotation.

## 4. Genome annotation

Repeat annotation was done by RepeatModeler (v2.0.2a) ^18^ with repeat masking by RepeatMasker (v4.1.2) ^19^. The sequence annotation used the repeat masked genome to annotate protein-coding genes using MAKER (v3.01.04) ^20^, with the assembled transcriptome as the EST evidence, and NCBI protein sequences of Decapoda species as the protein homology evidence. tRNAscan-SE (v2.0.12) ^21^ and RNAmmer (v1.2) ^22^ were employed to annotate tRNA and rRNA genes, respectively. Following sequence annotation, BLAST and funannotate (v1.8.13) were utilized for assigning putative functions and database IDs to the proteins.

## 5. *In silico* allergen identification

A set of the query sequences was created by downloading all shellfish allergen sequences, including Decapoda from Animalia Arthropoda and Animalia Mollusca on the WHO/IUIS Allergen Nomenclature database, to identify potential allergens in S. serrata. BLAST databases were built from the *S. serrata* genome assembly and annotation generated in steps 2 and 4 respectively for TBLASTN and BLASTP searches against the downloaded allergens. The *in silico* identification returned a list of putative *S. serrata* allergens exhibiting sequence homology to reported allergens.

With a substantial amount of Decapoda genomes and allergens, we specifically downloaded the whole genomes for species with allergens identified from the WHO/IUIS database. These included the NCBI RefSeq of *S*. *paramamosain* (Accession number: GCF_035594125.1), *Procambarus* (*P*.) *clarkii* (GCF_020424385.1), *Homarus* (*H.*) *americanus* (GCF_018991925.1), *Penaeus* (*P*.) *monodon* (GCF_015228065.2), *Eriocheir* (*E*.) *sinensis* (GCF_024679095.1), and *Macrobrachium* (*M*.) *nipponense* (GCF_015104395.2). Additionally, the GenBank files of *Chionoecetes* (*C.*) *opilio* (GCA_016584305.1) were downloaded. One outgroup species, *Hyalella (H.)* *azteca* (GCF_000764305.2) from the order Amphipoda, was also included.

Multiple alignment was done to study the homology of gene families between species by Clustal Omega (v1.2.4) ^23^. Allergen homologs were identified from BLAST, and their sequences were aligned with the MUSCLE algorithm. Maximum likelihood trees were constructed with bootstrap replicate 100 using MEGA11 (v11.0.13) ^24^. The phylogenetic tree was visualized using Interactive Tree of Life (iTOL) (v6.9) ^25^.

## 6. Gene expression level analysis

Salmon (v1.10.3) with the mapping-based mode was used to quantify the expression level of the putative allergens in *S. serrata* ^26^. An index was built with the coding sequences of the manually curated allergens and the *Gapdh* gene. The index was used to quantify the same 16 sets of paired-end transcriptome sequenced from six tissues of *S. serrata* (from transcriptome sequencing in step 3). The use of wild, adult samples ensures that the observed gene expression levels are representative of natural growth conditions of *S. serrata*, providing valuable insights into the allergenicity of mud crabs in their native habitat. The quantification returned the abundance level of each sequence as Transcripts per Million (TPM). The TPM was normalized using the value of the *Gadph* (normalized TPM, nTPM). The relative gene expression level was expressed in log_2_(nTPM + 1) and visualized in a heatmap generated by GraphPad Prism (v10.2) which is a comprehensive tissue-specific expression atlas for comparing the expression of allergens in different tissues of *S. serrata*.

## 7. Immunoassays

The coding sequences of several predicted allergens were optimized. The gene was synthesized and sub-cloned into the vector pET-30a(+) then transformed for protein expression. The recombinant protein with over 90% purity was validated by SDS-PAGE and Western blotting. On the other hand, total protein extracts from the same *S. serrata* were prepared from homogenized body muscles.

Serum samples were collected in two batches, with the first batch from allergic patients with informed consent at the First Affiliated Hospital of Guangzhou Medical University, while the second batch was collected from crab-allergic patients in the Prince of Wales Hospital in Hong Kong. The patients either have symptoms like asthma and atopic dermatitis, and/or had positive IgE results of ≥ 0.35 kU_A_/L by ImmunoCAP f23, crab (Thermo Fisher Scientific, USA). Crab-positive samples were screened using crab total proteins. Afterwards, indirect ELISA was performed for allergenicity testing with recombinant proteins. Proteins (5 μg/ml) diluted in coating buffer (100 mM Na2CO3·100 mM NaHCO3, pH=9.6) were applied to 96-well plates, with 37°C incubation for 3 hours. Then, the plate was washed with PBST (0.05% Tween-20/PBS) and blocked with 8% fetal bovine serum (blocking buffer) for 2 hours at room temperature. Serum samples, diluted 1:5 in blocking buffer, were added and incubated overnight at 4°C. After washing, anti-human IgE-HRP antibodies (1:1000) were applied for 30 minutes at room temperature, followed by TMB substrate for color development. The reaction was stopped with 0.1M sulfuric acid, and the allergenicity indicated by optial density (OD) at 450 nm was measured using a Multiskan GO spectrophotometer. Crab-negative control sera determined the cutoff for crab-positive samples. Statistical significance of the OD difference was assessed using unpaired two-tailed t-tests and the results were shown by column scatter plots in GraphPad Prism (v10.2). The positive reaction rate for each recombinant protein was computed for a clear visualization of the ELISA results.

## 8. Immunoblotting and mass spectrometry

For two-dimensional (2-D) gel electrophoresis, a new protein extract was prepared with homogenized body muscles of the same *S. serrata* used in the genomic and transcriptomic study. The two dimensions separated proteins based on isoelectric point (pI) by isoelectric focusing and molecular weight by SDS-PAGE. The gel electrophoresis was performed in duplicates, one was subjected to Coomassie Blue staining to visualize the proteins, and the other was transferred to the membranes for Western blot.

The membrane was incubated with the pooled sera from the allergic patients in the Guangzhou batch same as the samples for ELISA. The incubation was completed overnight at 4°C. Following incubation, the membrane was washed three times by TBST (TBS and 0.5% Tween-20), with each wash lasting for 10 minutes. Subsequently, the membrane was incubated with an anti-human IgE-HRP conjugate (SouthernBiotech, USA) diluted 1:2000 in blocking solution for one hour at room temperature. After six 10-minute washes with TBST, the membrane was treated with ECL chemiluminescent substrate solution (Solarbio, China) for detecting tagged proteins. The manually confirmed protein band on the immunoblot with corresponding spots found on the stained gel was cut precisely with a razor blade.

Each of the excised proteins was digested with trypsin for Liquid Chromatography–Mass Spectrometry (LC-MS/MS). The raw spectrum files were converted into formats for database search against the peptide sequences from deposited in protein databases constructed with the *in silico* proteome of *S. serrata* with MASCOT (v2.3.02) (Matrix Sciences, UK).

# References

1. Nonthawong K, Srisomsap C, Chokchaichamnankit D, Svasti J, Phiriyangkul P. Comparative proteomics and in silico allergenicity of fresh and powdered skipjack tuna and nile tilapia. *Food control*. 2023;144:109345. doi: 10.1016/j.foodcont.2022.109345.

2. Saetang J, Tipmanee V, Benjakul S. In silico prediction of cross-reactive epitopes of tropomyosin from shrimp and other arthropods involved in allergy. *Molecules (Basel, Switzerland)*. 2022;27(9):2667. <https://www.ncbi.nlm.nih.gov/pubmed/35566021>. doi: 10.3390/molecules27092667.

3. Kolmogorov M, Yuan J, Lin Y, Pevzner PA. Assembly of long, error-prone reads using repeat graphs. *Nature Biotechnology*. 2019;37(5):540–546. <https://www.ncbi.nlm.nih.gov/pubmed/30936562>. doi: 10.1038/s41587-019-0072-8.

4. Vaser R, Sović I, Nagarajan N, Šikić M. Fast and accurate de novo genome assembly from long uncorrected reads. *Genome Research*. 2017;27(5):737–746. <https://www.ncbi.nlm.nih.gov/pubmed/28100585>. doi: 10.1101/gr.214270.116.

5. Walker BJ, Abeel T, Shea T, et al. Pilon: An integrated tool for comprehensive microbial variant detection and genome assembly improvement. *PLoS ONE*. 2014;9(11):e–e112963. <https://www.ncbi.nlm.nih.gov/pubmed/25409509>. doi: 10.1371/journal.pone.0112963.

6. Boetzer M, Pirovano W. SSPACE-LongRead: Scaffolding bacterial draft genomes using long read sequence information. *BMC Bioinformatics*. 2014;15(1):211–211. <https://www.ncbi.nlm.nih.gov/pubmed/24950923>. doi: 10.1186/1471-2105-15-211.

7. Xu M, Guo L, Gu S, et al. TGS-GapCloser: A fast and accurate gap closer for large genomes with low coverage of error-prone long reads. *GigaScience*. 2020;9(9). <https://www.ncbi.nlm.nih.gov/pubmed/32893860>. doi: 10.1093/gigascience/giaa094.

8. Gurevich A, Saveliev V, Vyahhi N, Tesler G. QUAST: Quality assessment tool for genome assemblies. *Bioinformatics*. 2013;29(8):1072–1075. <https://www.ncbi.nlm.nih.gov/pubmed/23422339>. doi: 10.1093/bioinformatics/btt086.

9. Simão FA, Waterhouse RM, Ioannidis P, Kriventseva EV, Zdobnov EM. BUSCO: Assessing genome assembly and annotation completeness with single-copy orthologs. *Bioinformatics*. 2015;31(19):3210–3212. <https://www.ncbi.nlm.nih.gov/pubmed/26059717>. doi: 10.1093/bioinformatics/btv351.

10. Durand NC, Shamim MS, Machol I, et al. Juicer provides a one-click system for analyzing loop-resolution hi-C experiments. *Cell systems*. 2016;3(1):95–98. <https://dx.doi.org/10.1016/j.cels.2016.07.002>. doi: 10.1016/j.cels.2016.07.002.

11. Dudchenko O, Batra SS, Omer AD, et al. De novo assembly of the aedes aegypti genome using hi-C yields chromosome-length scaffolds. *Science*. 2017;356(6333):92–95. <https://www.jstor.org/stable/24918168>. doi: 10.1126/science.aal3327.

12. Durand NC, Robinson JT, Shamim MS, et al. Juicebox provides a visualization system for hi-C contact maps with unlimited zoom. *Cell systems*. 2016;3(1):99–101. <https://dx.doi.org/10.1016/j.cels.2015.07.012>. doi: 10.1016/j.cels.2015.07.012.

13. Luo R, Liu B, Xie Y, et al. SOAPdenovo2: An empirically improved memory-efficient short-read de novo assembler. *Gigascience*. 2015;4(1). <https://www.proquest.com/docview/3131615660>. doi: 10.1186/2047-217X-1-18.

14. Daehwan Kim, Joseph M. Paggi, Chanhee Park, Christopher Bennett, Steven L. Salzberg. Graph-based genome alignment and genotyping with HISAT2 and HISAT-genotype. *Nature Biotechnology*. 2019;37(8):907–915. doi: 10.1038/s41587-019-0201-4.

15. Pertea M, Pertea GM, Antonescu CM, Chang T, Mendell JT, Salzberg SL. StringTie enables improved reconstruction of a transcriptome from RNA-seq reads. *Nature Biotechnology*. 2015;33(3):290–295. <https://www.ncbi.nlm.nih.gov/pubmed/25690850>. doi: 10.1038/nbt.3122.

16. Pertea G, Pertea M. GFF utilities: GffRead and GffCompare. *F1000 research*. 2020;9:304. <https://www.ncbi.nlm.nih.gov/pubmed/32489650>. doi: 10.12688/f1000research.23297.1.

17. Friedman N, Regev A, Grabherr MG, et al. Full-length transcriptome assembly from RNA-seq data without a reference genome. *Nature Biotechnology*. 2011;29(7):644–652. <http://dx.doi.org/10.1038/nbt.1883>. doi: 10.1038/nbt.1883.

18. Flynn JM, Hubley R, Goubert C, et al. RepeatModeler2 for automated genomic discovery of transposable element families. *Proceedings of the National Academy of Sciences*. 2020;117(17):9451–9457. <https://www.jstor.org/stable/26929951>. doi: 10.1073/pnas.1921046117.

19. Tarailo-Graovac M, Chen N. Using RepeatMasker to identify repetitive elements in genomic sequences. *Current Protocols in Bioinformatics*. 2009;25(1):4.10.1–4.10.14. <https://onlinelibrary.wiley.com/doi/abs/10.1002/0471250953.bi0410s25>. doi: 10.1002/0471250953.bi0410s25.

20. Campbell MS, Holt C, Moore B, Yandell M. Genome annotation and curation using MAKER and MAKER‐P. *Current Protocols in Bioinformatics*. 2014;48(1):1–4.11.39. <https://onlinelibrary.wiley.com/doi/abs/10.1002/0471250953.bi0411s48>. doi: 10.1002/0471250953.bi0411s48.

21. Chan P, Lin B, Mak A, Lowe T. tRNAscan-SE 2.0: Improved detection and functional classification of transfer RNA genes. *Nucleic Acids Research*. 2021;49(16):9077–9096. <https://search.proquest.com/docview/2563421373>. doi: 10.1093/nar/gkab688.

22. Lagesen K, Hallin P, Rødland EA, Stærfeldt H, Rognes T, Ussery DW. RNAmmer: Consistent and rapid annotation of ribosomal RNA genes. *Nucleic Acids Research*. 2007;35(9):3100–3108. <https://www.ncbi.nlm.nih.gov/pubmed/17452365>. doi: 10.1093/nar/gkm160.

23. Madeira F, Pearce M, Tivey ARN, et al. Search and sequence analysis tools services from EMBL-EBI in 2022. *Nucleic Acids Research*. 2022;50(W1):276. <https://www.ncbi.nlm.nih.gov/pubmed/35412617>. doi: 10.1093/nar/gkac240.

24. Tamura K, Stecher G, Kumar S. MEGA11: Molecular evolutionary genetics analysis version 11. *Molecular Biology and Evolution*. 2021;38(7):3022–3027. <https://www.ncbi.nlm.nih.gov/pubmed/33892491>. doi: 10.1093/molbev/msab120.

25. Letunic I, Bork P. Interactive tree of life (iTOL) v6: Recent updates to the phylogenetic tree display and annotation tool. *Nucleic Acids Research*. 2024. <https://www.ncbi.nlm.nih.gov/pubmed/38613393>. doi: 10.1093/nar/gkae268.

26. Patro R, Duggal G, Love MI, Irizarry RA, Kingsford C. Salmon provides fast and bias-aware quantification of transcript expression. *Nature methods*. 2017;14(4):417–419. <https://www.ncbi.nlm.nih.gov/pubmed/28263959>. doi: 10.1038/nmeth.4197.
